# Alpha-Melanocyte-Stimulating Hormone and Agouti-Related Protein: Do They Play a Role in Appetite Regulation in Childhood Obesity?

**DOI:** 10.4274/jcrpe.2136

**Published:** 2016-03-01

**Authors:** Aysel Vehapoğlu, Serdar Türkmen, Şule Terzioğlu

**Affiliations:** 1 Bezmialem Vakıf University Faculty of Medicine, Department of Pediatrics, İstanbul, Turkey; 2 Gaziosmanpaşa Taksim Training and Research Hospital, Clinic of Biochemistry, İstanbul, Turkey; 3 Bezmialem Vakıf University Faculty of Medicine, Department of Medicinal Biology, İstanbul, Turkey

**Keywords:** Alpha-melanocyte-stimulating hormone, agouti-related protein, underweight, Childhood obesity

## Abstract

**Objective:**

The hypothalamus plays a crucial role in the regulation of feeding behavior. The anorexigenic neuropeptide alpha-melanocyte-stimulating hormone (α-MSH) and the orexigenic neuropeptide agouti-related protein (AgRP) are among the major peptides produced in the hypothalamus. This study investigated the plasma concentrations of α-MSH and AgRP in underweight and obese children and their healthy peers. The associations between α-MSH and AgRP levels and anthropometric and nutritional markers of malnutrition and obesity were also assessed.

**Methods:**

Healthy sex-matched subjects aged 2 to 12 years were divided into 3 groups, as underweight (n=57), obese (n=61), and of normal weight (n=57). Plasma fasting concentrations of α-MSH and AgRP were measured by enzyme-linked immunosorbent assay. The differences between the three groups as to the relationships between plasma concentrations of α-MSH and AgRP and anthropometric data, serum biochemical parameters and homeostatic model assessment of insulin resistance were evaluated.

**Results:**

Obese children had significantly lower α-MSH levels than underweight (1194±865 vs. 1904±1312 ng/mL, p=0.006) and normal weight (1194±865 vs. 1762±1463 ng/mL, p=0.036) children; there were no significant differences in the α-MSH levels between the underweight and normal weight children (p=0.811). Also, no significant differences were observed between the underweight and obese children regarding the AgRP levels (742±352 vs. 828±417 ng/mL, p=0.125). We found a significant positive correlation between plasma α-MSH and AgRP levels across the entire sample.

**Conclusion:**

This study is the first to demonstrate body weight-related differences in α-MSH and AgRP levels in children. Circulating plasma α-MSH levels in obese children were markedly lower than those of underweight and normal-weight children. This suggests that α-MSH could play a role in appetite regulation.

**WHAT IS ALREADY KNOWN ON THIS TOPIC?**

Children require sufficient nutrients to support the immune system and to help the body maintain health and normal bodily functions. Appetitive hormones are of interest in human populations because they are implicated in appetite regulation, weight loss and gain, malnutrition, and obesity. Circulating levels of alpha-melanocyte-stimulating hormone (α-MSH) and agouti-related protein (AgRP), and the potential role of these proteins in childhood malnutrition and obesity, have not yet been studied; scant data exist regarding their circulatory function in children.

**WHAT THIS STUDY ADDS?**

We assessed the peripheral concentrations of α-MSH and AgRP in three different groups (underweight and obese children and their healthy peers) and investigated the changes in the levels of these peptides with respect to body mass index, insulin, and homeostatic model assessment of insulin resistance. We hope this study will be useful for our colleagues.

## INTRODUCTION

Two major public health problems in the adult population that involve energy balance, namely obesity and anorexia, also appear in childhood. Body weight and fat levels are low in anorexic children who eat slowly, consume a limited number of foods, lack interest in food, display an irregular eating pattern, and experience loss of appetite and less frequent hunger episodes (1). Obesity, a multifactorial disorder resulting from the interactions among genetic, psychological, physical, environmental, and socioeconomic factors, develops only if energy intake from feeding chronically exceeds total energy expenditure. Feeding behavior is regulated by a system, with the hypothalamus at its center, in which the amount eaten is determined by the body’s response to the internal energy status (2). There are complex interconnections between the hypothalamic nuclei that maintain energy homeostasis by regulating food intake and energy expenditure; the latter includes physical activity, basal metabolism, and adaptive thermogenesis (3,4).

One of the major regulators of food intake is leptin, a hormone released by adipose tissue that induces satiety via receptors located in the arcuate nucleus. Leptin crosses the blood-brain barrier and acts directly on two populations of neurons within the arcuate nucleus that express agouti-related protein (AgRP) or proopiomelanocortin (POMC). The POMC system plays a crucial role in the regulation of feeding behavior. Alpha-melanocyte-stimulating hormone (α-MSH) is a potent anorexigenic neuropeptide (5); leptin stimulates the production of α-MSH, which is an agonist for melanocortin-4 receptors (MC4R) and melanocortin-3 receptors (MC3R), and inhibits the production of AgRP (an antagonist for these receptors) in a coordinated manner to regulate the energy balance by inhibiting food intake and stimulating energy expenditure (6).

Recent research indicates that α-MSH is produced in the human pituitary by cells of the pars distalis of the pituitary gland and numerous extrapituitary cells, including monocytes, astrocytes, gastrointestinal cells, and keratinocytes (7). AgRP is among the most potent and long-lasting appetite stimulators and exerts its effects primarily by opposing the anorexigenic/catabolic actions of POMC by competitively inhibiting melanocortin receptors (specifically MC3-R and MC4-R) at the postsynaptic level. It is encoded by the AgRP gene. AgRP expression has been detected in a range of human tissues including the brain, adrenal glands, testis, lung, and kidney. α-MSH plays a role in thermal regulation (hypermetabolic/hyperthermic) by increasing free fatty acid oxidation in skeletal muscle (8,9). Five melanocortin receptors are known; two of these, namely, MC3-R and MC4-R, are believed to be involved in energy balance signaling. Mutations in MC4-R have been shown to play a major role in the genetics of obesity (10). However, circulating levels of MSH and AgRP, and the potential role of these proteins in childhood malnutrition and obesity, have not yet been studied; scant data exist regarding their effects on circulatory function in children. Taking the information listed above into consideration, we aimed to assess the peripheral concentrations of α-MSH and AgRP in three groups (underweight and obese children and their healthy peers) and to investigate the differences in the levels of these peptides with respect to body mass index (BMI), insulin, and homeostatic model assessment of insulin resistance (HOMA-IR).

## METHODS

This cross-sectional study was conducted at the Bezmialem Vakıf University Hospital in İstanbul, on randomly selected pediatric patients who attended the outpatient clinic of the pediatrics department for routine examinations between October 2014 and March 2015. The study groups comprised 57 underweight prepubertal children (26 males and 31 females with a BMI for age and sex <18.5th percentile due to loss of appetite and infrequent hunger episodes), 61 obese children (28 males and 33 females with a BMI for age and sex ≥95th percentile), and 57 healthy children of normal weight (26 males and 31 females with a BMI for age and sex between the 18.5th to 95th percentiles). The inclusion criterion did not comprise a dietary history of inadequate nutrient intake (in quality or quantity). Only children with no health problems, except for anorexia or obesity, were included in the study. The underweight patients had the opportunity to obtain food, but had poor appetite; they were diagnosed with malnutrition on admission, but no patient was receiving therapy or nutritional support during the evaluation. The exclusion criteria included presence of an endocrine disease or a syndromal problem, of an acute or chronic inflammatory disease, a malabsorption syndrome such as celiac disease or cystic fibrosis. Presence of an infectious or systemic disease, use of prescription medications, vitamins, or mineral supplements for any reason, were also reasons for exclusion. None of the subjects had a history or any current evidence of metabolic, cardiovascular, or hepatic disease.

Anthropometric measurements were performed for all patients; height was measured to the nearest 0.1 cm using a Harpenden fixed stadiometer, and weight was measured to the nearest 0.1 kg using a SECA balance scale with the subject dressed only in light underwear without shoes. Using this information, age- and sex-specific BMI percentiles were calculated. BMI expresses the relationship between weight and height as a ratio (weight in kg divided by height in m2) and is strongly correlated with the percent body fat. In the present study, childhood BMI was calculated as described above and the subjects were classified as thin, normal, or obese according to Cole’s recently published BMI cut-offs for children aged 2 to 18 years for thinness/overweight/obese according to sex and age (11).

After 12 hours of fasting, venous blood samples were collected into tubes (Vacuette; Greiner Labor Technic GmbH, Germany) between 8:00 am and 9:00 am The samples were then separated by centrifugation (10 min at 4500 rpm, 4 °C) and stored at -80 °C until subsequent use. A complete blood count, serum iron level, serum total iron-binding capacity, ferritin, thyroid stimulating hormone, thyroxine, vitamin B12, folic acid, glucose, albumin, insulin, total immunoglobulin A (IgA), tissue transglutaminase antibody IgA (anti tTG-IgA), total cholesterol, triglycerides, C-reactive protein (CRP), plasma α-MSH and AgRP levels, and erythrocyte sedimentation rate were assessed in all subjects. Insulin resistance (IR) was estimated from fasting plasma measurements using HOMA-IR [insulin (mU/L)×glucose (mmol/L)/22.5]. The criterion for IR in prepubertal children is a HOMA-IR of >2.5 (12). Total cholesterol and triglycerides were measured using the homogeneous colorimetric enzyme technique (Roche Cobas 8000 modular analyzer; Roche Diagnostics, Mannheim, Germany). Glucose was measured using the glucose oxidase technique (Advia 1800; Siemens Healthcare Diagnostics, Tarrytown, NY, USA); insulin levels were analyzed using the direct chemiluminescence technique (Advia Centaur, Siemens). Plasma α-MSH and AgRP concentrations were measured using commercially available enzyme-linked immunosorbent assay kits purchased from SunRed (SRB/Shanghai; cat no. 201-12-5500; sensitivity: 14.068 ng/L, range: 15-4200 ng/L, intraassay CV, 7.4%; cat no. 201-12-1479, sensitivity: 4.776 ng/L, range: 5-1500 ng/L, intraassay CV, 4.3%, respectively) according to the manufacturer’s protocol. Standards and samples were incubated with antibody-coated 96-well plates for 2 hours. Enzyme-linked antibodies for the proteins were then incubated for 1 hour. Finally, the substrate solution was added; the reaction stopped after a short while. The intensity of the color in each well was measured using a microplate reader (VarioskanTM Flash Multimode Reader; Thermo Scientific, Hudson, NH, USA) at 450 nm.

Analyses were conducted using the IBM Statistical Package for the Social Sciences for Windows software package (version 20.0; IBM Corp., Armonk, NY, USA). The results are presented as means ± standard deviation (SD), with categorical variables presented as frequencies and percentages. Comparison of group means was performed using one-way ANOVA for parametric tests with Tukey’s honestly significant difference (HSD) post-hoc test applied for multiple comparisons. Pearson’s correlation was used to determine relationships between variables. Categorical data were compared using the chi-squared test. A p-value of <0.05 was taken to indicate statistical significance.

The study protocol was carried out in accordance with the ethical principles of the Declaration of Helsinki, 1989. Information concerning the aim of the study was provided to the children’s parents at the time of enrollment; written informed consent was also obtained. Ethical approval was granted by the Bezmialem Vakıf University Local Research Ethics Committee.

## RESULTS

The experimental groups were randomly selected and comprised prepubertal underweight children (n=57; 26 males and 31 females, mean age: 7.7±2.4 years, range: 2-12 years) with a mean ± SD score for BMI of -1.91±0.7 (thinness grade of 1-3); obese children (n=61; 28 males and 33 females, mean age: 8.1±2.2 years, range: 2-12 years) with a mean ± SD score for BMI of 2.35±0.6; and healthy children of normal weight (n=57; 26 males and 31 females, mean age: 7.4±2.7 years, range: 2-12 years) with a mean ± SD scores for BMI of 0.26±0.8. In the underweight group, 32 (56.1%) subjects had a thinness grade of 1, 20 (35.0%) had a thinness grade of 2, and 5 (8.7%) had a thinness grade of 3. The anthropometric and metabolic characteristics of the three groups are summarized in Tables 1 and 2. The age and sex distribution did not differ among the groups (p=0.720 and 0.999, respectively). The underweight group had a significantly lower mean weight, weight z-score, mean height, height z-score, BMI, BMI z-score, and BMI percentage as compared to the control and obese groups (p<0.001). There were no significant group differences in the hemoglobin, albumin or ferritin levels (p=0.770, 0.680, 0.409, respectively). There were no significant differences between the underweight and normal-weight children in fasting glucose, insulin, total cholesterol, triglyceride, CRP or HOMA-IR (p=0.993, 0.247, 0.913, 0.494, 0.999, and 0.391, respectively) levels. The obese group had higher serum fasting glucose, insulin, total cholesterol, triglycerides, CRP and HOMA-IR than did the other two groups (p<0.001). HOMA-IR was positively correlated with glucose and insulin levels in all groups (all p<0.001). The level of vitamin B12 was higher in normal-weight children than in underweight or obese children (p=0.021 and 0.030, respectively), but there were no differences between the underweight and obese children. In the obese group, 46 (75%) subjects had a HOMA-IR of >2.5.

**Figure 1:**
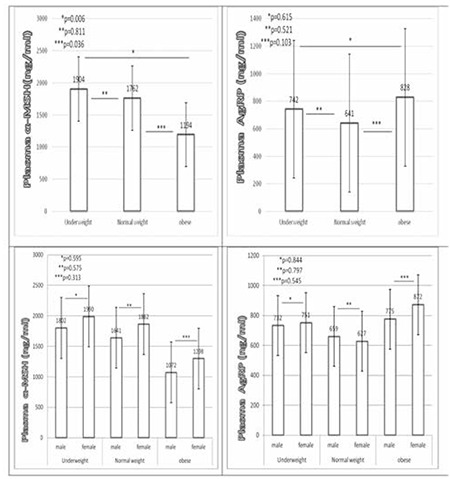
Plasma alpha-melanocyte-stimulating hormone and agouti-related protein levels in underweight, normal-weight, and obese children. The data are expressed as mean values ± standard error of mean. One-way ANOVA followed by Post Hoc Tukey’s test was used for correlation analysis; p<0.05

Obese children had significantly lower α-MSH levels than did the underweight (1194±865 vs. 1904±1312 ng/mL, p=0.006) and normal-weight (1194±865 vs. 1762±1463 ng/mL, p=0.036) children; there were no significant differences in α-MSH levels between the underweight and normal-weight children (p=0.811). There were also no significant differences between the underweight and obese children regarding the AgRP levels (742±352 vs. 828±417, p=0.125) (Table 3). Furthermore, the plasma AGRP and α-MSH levels were not significantly different between males and females across the entire sample (Figure 1).

There was a significant positive correlation between plasma α-MSH and AgRP levels across the entire sample. The results of Pearson’s correlation of α-MSH, AgRP, and HOMA-IR with age, glucose and insulin levels are summarized in Table 4. When the entire sample was evaluated together (n=175), there was a significant positive correlation between the plasma α-MSH and AgRP levels (p<0.001) and a negative correlation between the α-MSH levels and weight z-score (p=0.017), BMI (p=0.019), BMI z-score (p=0.014), BMI percentile (p=0.017), insulin (p=0.045), and CRP (p<0.001).

## DISCUSSION

Appetitive hormones are of interest in human populations because they are implicated in appetite regulation, weight loss and gain, malnutrition, and obesity. There are several reports on plasma α-MSH and AgRP levels in adults and children (13,14). The present study demonstrated that circulating plasma α-MSH levels in obese children are markedly lower than those in underweight and normal-weight children. We also found that AgRP levels were relatively lower in underweight children than in controls, but not significantly. We found no significant differences between females and males in α-MSH or AgRP levels. When the entire sample was evaluated together, α-MSH levels were negatively correlated with various parameters of obesity, including weight z-score, BMI, BMI z-score, BMI percentile, as well as insulin and CRP levels.

As the central feeding organ, the hypothalamus mediates the regulation of short and long-term dietary intake via synthesis of orexigenic and anorectic neuropeptides. Previous studies in animals demonstrated that α-MSH in the peripheral circulation plays a role in metabolism regulation, fat storage, and glucagon secretion (15,16). Studies investigating the role of peripheral α-MSH and AgRP in humans have reported equivocal results. Hoggard et al (17) showed that both plasma α-MSH and AgRP levels were elevated in 18 obese subjects relative to 11 lean adults; in both cases there was also a close correlation with both BMI and body fat mass. Furthermore, there were no significant changes in plasma α-MSH levels in response to either food deprivation or food restriction in either the lean or obese subjects. Similarly, Katsuki et al (18) demonstrated that both plasma α-MSH and AgRP levels were higher in obese than non-obese men; furthermore, plasma AgRP levels were significantly correlated with plasma α-MSH levels, suggesting a degree of peripheral involvement in energy balance regulation. Another study found that plasma α-MSH levels were similar between normal-weight, overweight, and obese subjects, although there was a weak trend toward higher α-MSH levels in obese than in lean men (19). In contrast, Nam et al (20) found that plasma and cerebrospinal α-MSH levels in obese women did not differ significantly from those of controls at baseline or after a 5% weight loss. Gavrila et al (21) demonstrated no relationship between peripheral α-MSH levels and body composition parameters in 108 healthy, normal-weight adults. We encountered only one comparable study of plasma α-MSH levels in children published previously (22). In this study, Roth et al (22) reported no significant differences in α-MSH levels between obese and normal-weight children, although children with craniopharyngioma had lower α-MSH levels than did obese and lean children. Furthermore, the authors found no significant differences in the α-MSH levels between females and males, similar to our study.

α-MSH binds to hypothalamic neurons that express MC4-R, leading to appetite suppression (23). Despite the fact that α-MSH is an anorexigenic neuropeptide, as mentioned above, many studies on adults have demonstrated that obese subjects had higher levels of α-MSH than do normal-weight or lean subjects. In contrast, our data showed that circulating plasma α-MSH levels in obese children were markedly lower than those in underweight and normal-weight children, which suggests that α-MSH plays a role in appetite regulation. More frequent hunger episodes due to α-MSH deficiency could lead to increased feeding signals, which may in turn increase the risk of developing obesity.

In the present study, when all three groups were analyzed together, there were significant negative correlations between α-MSH levels and parameters including the weight z-score, BMI, BMI z-score, BMI percentile, and insulin and CRP levels. These results suggest that α-MSH levels are probably more closely related to weight status in children, supporting a probable role of α-MSH in the peripheral regulation of energy homeostasis. Thus, it would appear that α-MSH levels decrease with increased BMI in children, which leads to diminished appetite suppression. Whether the observed differences are due to the more accurate effect estimates due to the relatively larger sample size of this study or to genetic differences in the studied populations requires further investigation.

The hypothalamic melanocortin system, which comprises the MC4-R, its agonist α-MSH, and its antagonist AgRP, is considered to be the main central anorexigenic pathway controlling energy homeostasis (24). Attention has focused particularly on the roles of peripheral α-MSH and AgRP in obesity, and several studies have reported on the peripheral actions of α-MSH and AgRP in the pathophysiology of eating disorders in adults (25). We found no previous studies on the levels of these proteins in underweight children, but studies in patients with anorexia nervosa suggest abnormal expression of appetite-regulating hormones. Moriya et al (26) showed that plasma AgRP levels were significantly higher in patients with anorexia nervosa than in controls, whereas plasma α-MSH levels did not differ between the two groups. These authors suggested that elevated plasma AgRP may be related to energy homeostasis disturbances in anorexia nervosa. Furthermore, a previous study showed that patients with acute anorexia nervosa had higher AgRP levels than those of healthy controls, but the AgRP levels of weight-restored anorexia patients were similar to those of healthy controls (27). In our study, no differences were found between obese and underweight children with respect to AgRP levels.

In patients with anorexia nervosa, decreased food intake is accompanied by hyperactivity and activation of the hypothalamic-pituitary-adrenal axis. Polymorphism in the gene encoding AgRP is associated with the development of both anorexia nervosa and obesity (28). Vink et al (29) suggested that genetic defects resulting in chronic activation of the melanocortinergic system could lead to anorexia nervosa. Variations in AgRP could be due to suppression of MC4-R, leading to a decreased feeding signal and thereby increasing the risk of developing anorexia nervosa. Interestingly, synthetic MC4-R antagonists, which act as AgRP mimetics, relieve different kinds of anorectic conditions. MC4-R antagonists have also been shown to be effective against cancer-induced anorexia (30). In the present study, our anorexic underweight children (due to loss of appetite and infrequent hunger episodes) had lower AgRP levels than their obese peers, although not significantly. Recently, there has been a growing interest in a number of genes related to appetite regulation (31). The importance of the melanocortin signaling pathway in humans for the control of appetite and energy balance is suggested by numerous monogenetic mutations identified in genes involved in the synthesis or processing of the glycoprotein POMC, or in mutations that lead to defects in POMC signaling via the melanocortin receptors. In particular, MC4-R mutations result in a severely obese phenotype and may be responsible for up to 4% of all cases of severe obesity in certain populations (31,32,33).

However, previous studies have also shown that peripheral α-MSH possesses a number of functions, such as anti-inflammatory and antimicrobial effects, and probably contributes to innate immunity. Recent research has shown that α-MSH is produced in the human pituitary by cells of the pars distalis and by numerous extrapituitary cells including monocytes, astrocytes, gastrointestinal cells, and keratinocytes (34). Human studies have demonstrated significant changes in endogenous peptide levels in pathological states. α-MSH is extremely effective in the treatment of animal models of local and systemic inflammatory disorders, including sepsis syndrome, adult respiratory distress syndrome, respiratory arrest, rheumatoid arthritis, inflammatory bowel disease, and encephalitis (35,36). Obese children have been shown to have elevated levels of inflammatory markers, of which CRP is strongly and positively associated with weight status in children (37). Our study demonstrated that obese children had higher CRP levels than their normal-weight and underweight peers; there was also a negative correlation between α-MSH and CRP levels, which suggests that a decreased anti-inflammatory effect of α-MSH in obese children increases the risk of elevated CRP levels.

The present study showed for the first time that circulating plasma α-MSH levels are lower in obese children than in underweight and normal-weight children and that they are also negatively correlated with body weight. The study also demonstrated that AgRP levels were lower in underweight children than in controls, but not significantly so. Decreased α-MSH levels in obese children appear to be important for understanding the physiology of energy homeostasis; further research in this area may lead to the development of novel treatment strategies for pediatric malnutrition and obesity. Neuropeptides that are involved in appetite regulation and energy expenditure could be important in future weight loss interventions.

## Figures and Tables

**Table 1:**
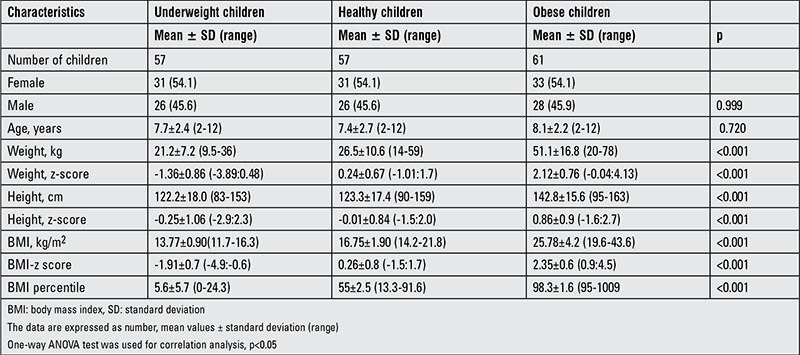
Comparison of demographic characteristics of underweight, normal weight and obese children

**Table 2:**
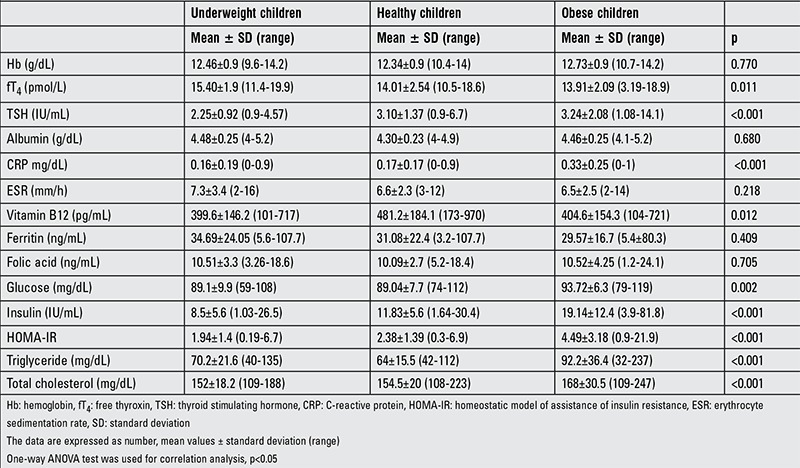
Laboratory findings in underweight, normal-weight, and obese children

**Table 3:**
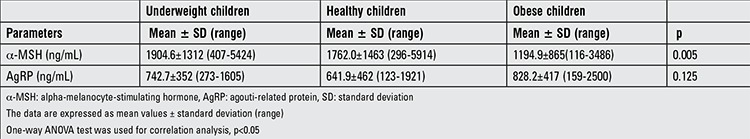
Study parameters of underweight, normal-weight, and obese children

**Table 4:**
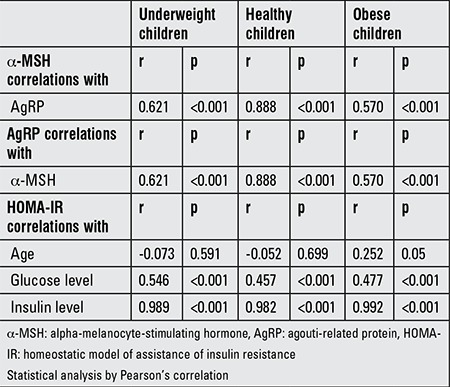
The results of Pearson’s correlation of alpha-melanocyte-stimulating hormone, agouti-related protein and homeostatic model of assistance of insulin resistance with age, glucose, and insulin levels between the groups
